# Supplementary material for: PARKIN is not required to sustain OXPHOS function in adult mammalian tissues
Source: NPJ Parkinsons Dis. 2024 Apr 29;10:93. doi: 10.1038/s41531-024-00707-0 (PMC11058849; doi:10.1038/s41531-024-00707-0)

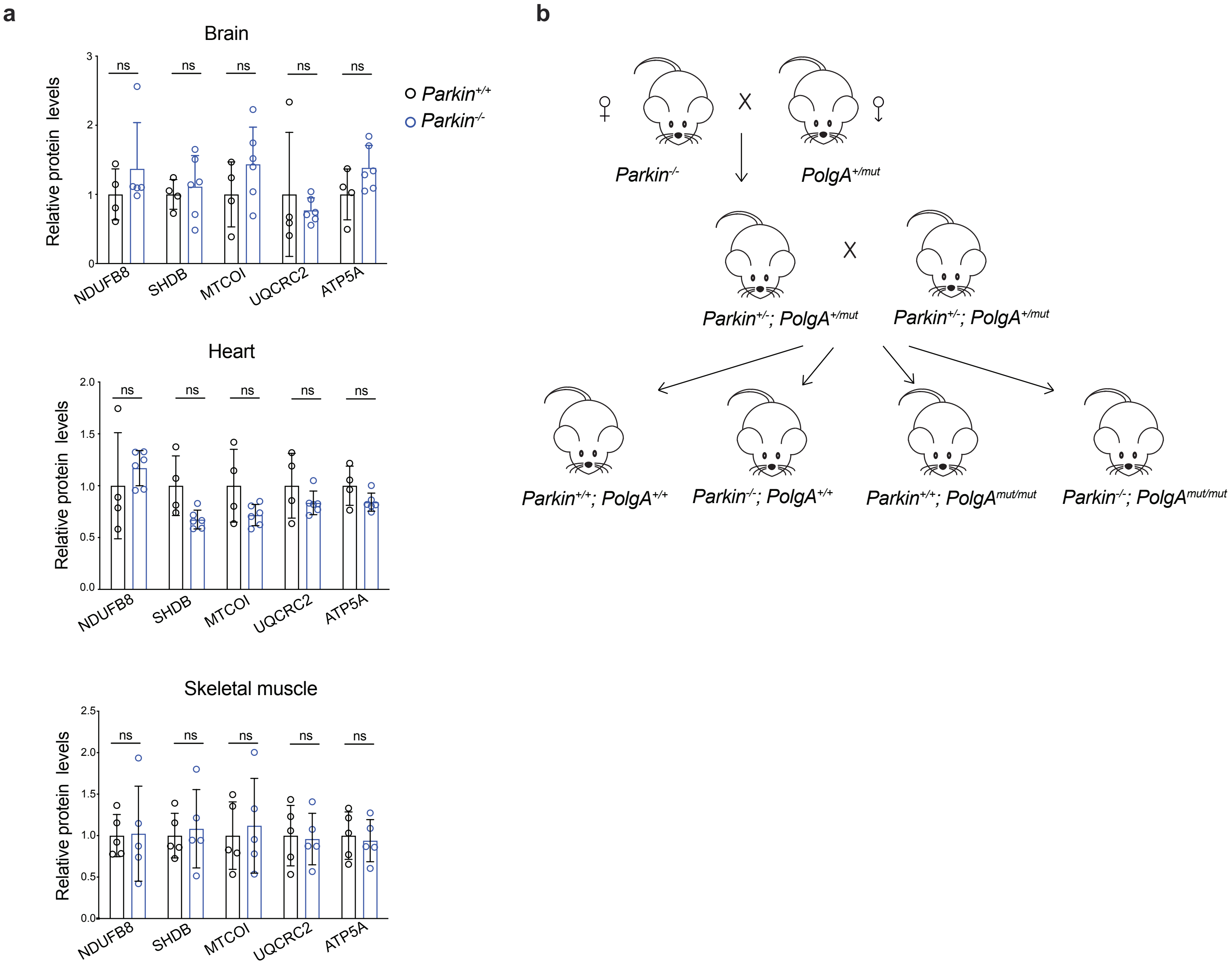

Supplementary Fig. 1. Ablation of *Parkin* in mouse models. a. Densitometric quantification of the steady-state levels of OXPHOS subunits in brain, heart, and skeletal muscle as determined by Western blots. Data are represented as mean ± s.e.m.; n≥4; ns= not significant. **b.** Breeding strategy used to generate the transgenic mouse line homozygous for both the whole-body *Parkin* knockout allele and the mtDNA mutator allele.

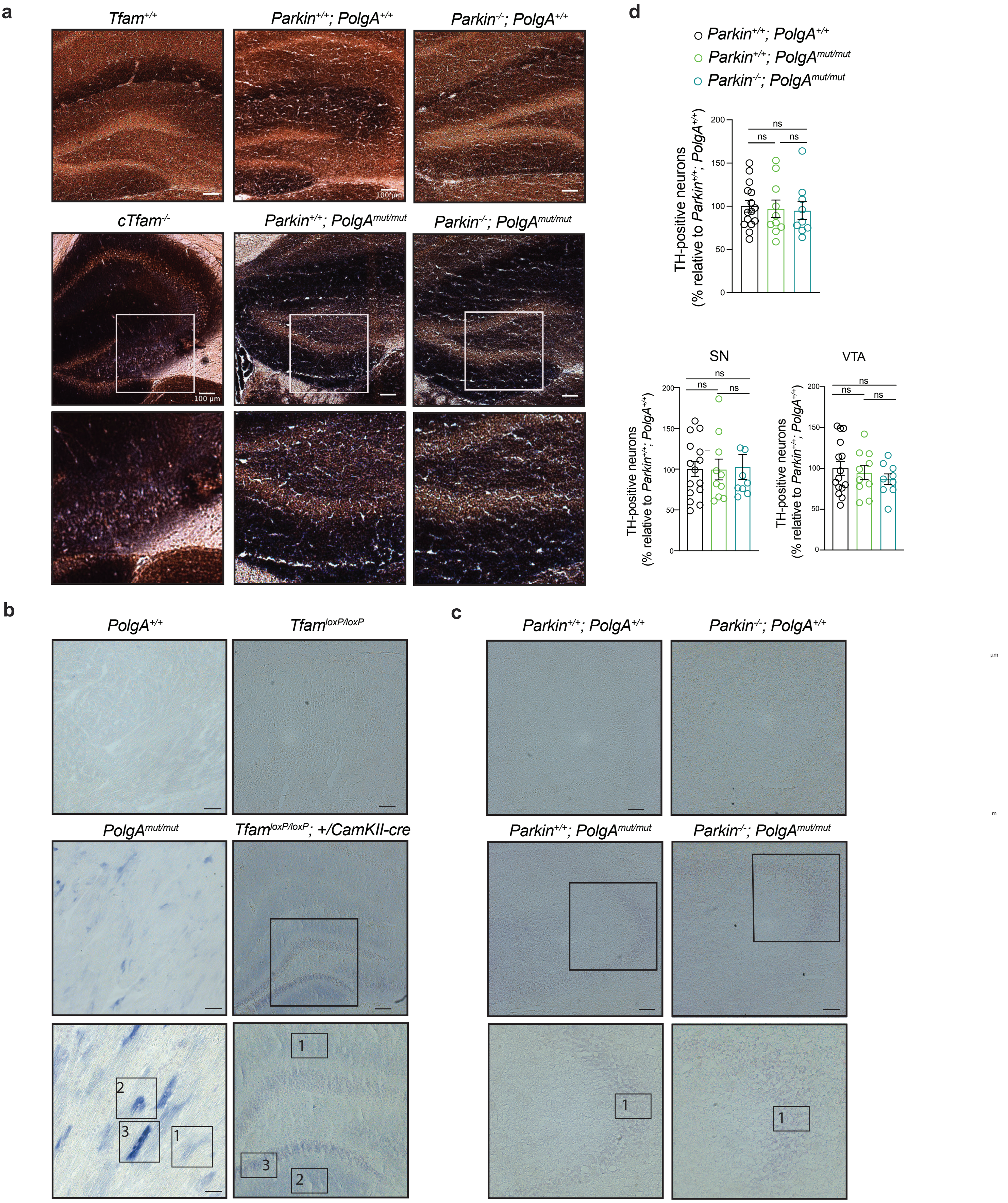

Supplementary Fig.2. COX activity of *Parkin$^{-/-}$; PolgA$^{mut/mut}$* mice. COX enzyme activity measured using **a.** COX/SDH in brain sections of MILON mice (conditional *Tfam* KO in forebrain neurons, *Tfam$^{loxP/loxP}$; +/CamKII-cre*) at 5 months of age and in hippocampal section of *Parkin* KO mice carrying WT mtDNA (*Parkin$^{-/-}$; PolgA$^{+/+}$*) or mutant mtDNA (*Parkin$^{-/-}$; PolgA$^{mut/mut}$*) . NBTx staining in **b.** the heart of mtDNA mutator mice (*PolgA$^{mut/mut}$*), which was used as positive controls with different levels of COX deficiency (1 to 3), and **c.** in the hippocampal section of *Parkin* KO mice carrying WT mtDNA (*Parkin$^{-/-}$; PolgA$^{+/+}$*) or mutant mtDNA (*Parkin$^{-/-}$; PolgA$^{mut/mut}$*). (Scale bars: 500 µm, 100 µm and 50 µm). **d.** Quantification of TH-positive DA neurons in ventral midbrain (SN and VTA) performed in midbrain sections using Cell Counter plugin. Data are represented as mean ± s.e.m.; n≥5 slices from 2-3 mice per genotype; ns= not significant.

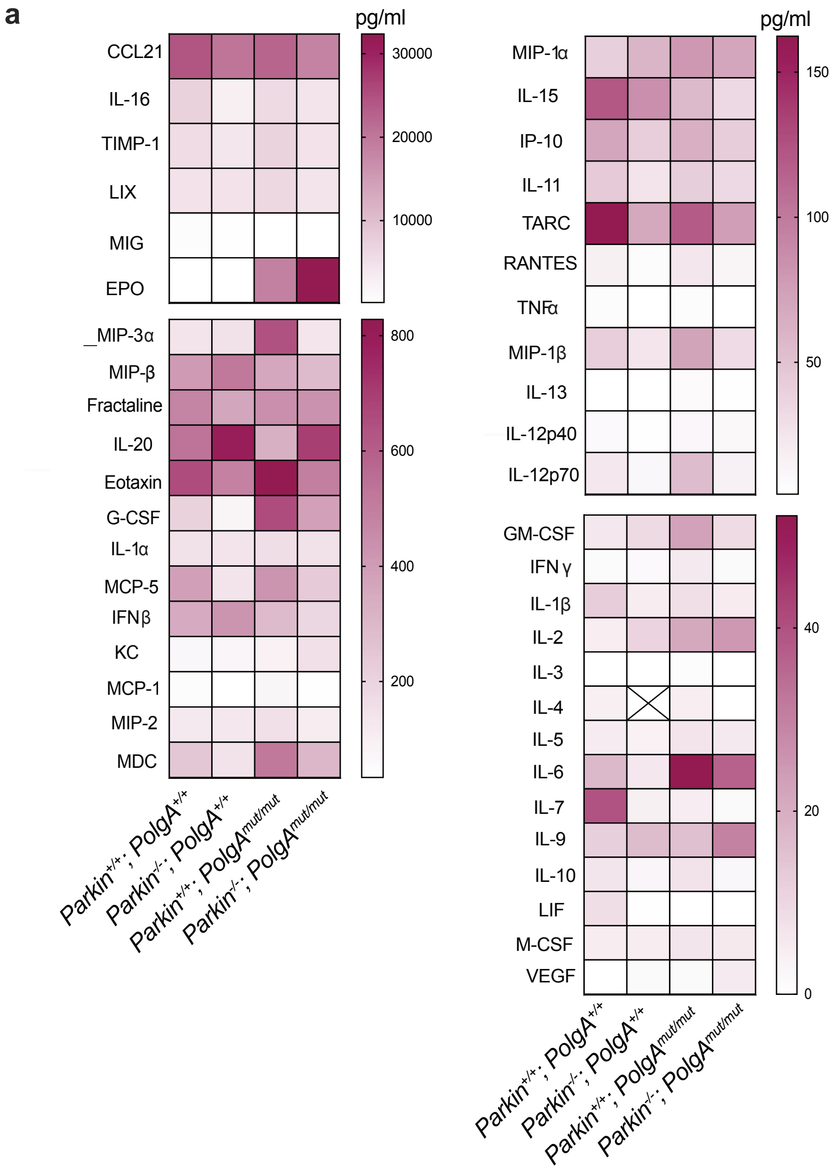

Supplementary Fig.3. Levels of pro-inflammatory mediators in *Parkin* $^{-/-}$*; PolgA* $^{mut/mut}$ mice. a. Heatmaps showing the levels (pg/ml) of 45 inflammatory cytokines and chemokines in plasma samples collected from 36-week-old mice; n ≥5 per genotype.

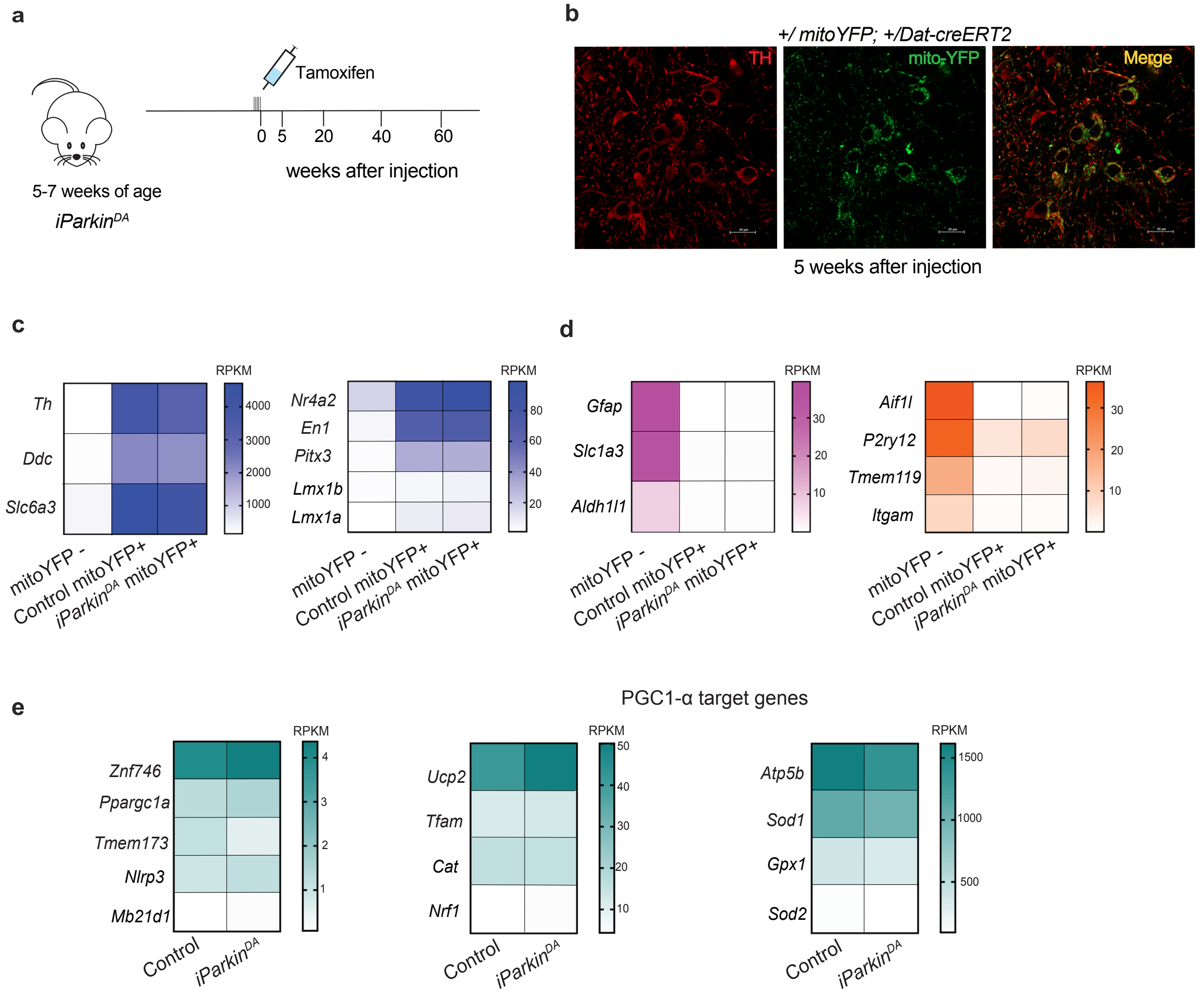

Supplementary Fig.4. Induction of PARKIN loss in adult DA neurons and transcriptomics analysis at 5 weeks after tamoxifen injection. a. Diagram depicting tamoxifen-induced inactivation of *Parkin* gene in adult mice. Mice at 5–7 weeks of age were intraperitoneally injected with tamoxifen for 5 consecutive days and examined up to 60 weeks after injection. **b.** Representative confocal microscopy images of mitoYFP-labelled mitochondria (green) in TH immuno-reactive neurons (red) at 5 weeks after tamoxifen injection (Scale bar: 20 μm). **c-d.** Heatmaps showing the expression levels of genes encoding **c.** DA neuronal markers, **d.** astrocyte and microglial markers in mitoYFP+ and mitoYFP- samples (Reads Per Kilobase Million, RPKM) at 5 weeks after injection. **e.** Heatmap showing the expression of genes involved in mitochondrial biogenesis and inflammation in mitoYFP+ cells isolated from *iParkinDA* and control mice at 5 weeks after injection. n>5 per genotype.

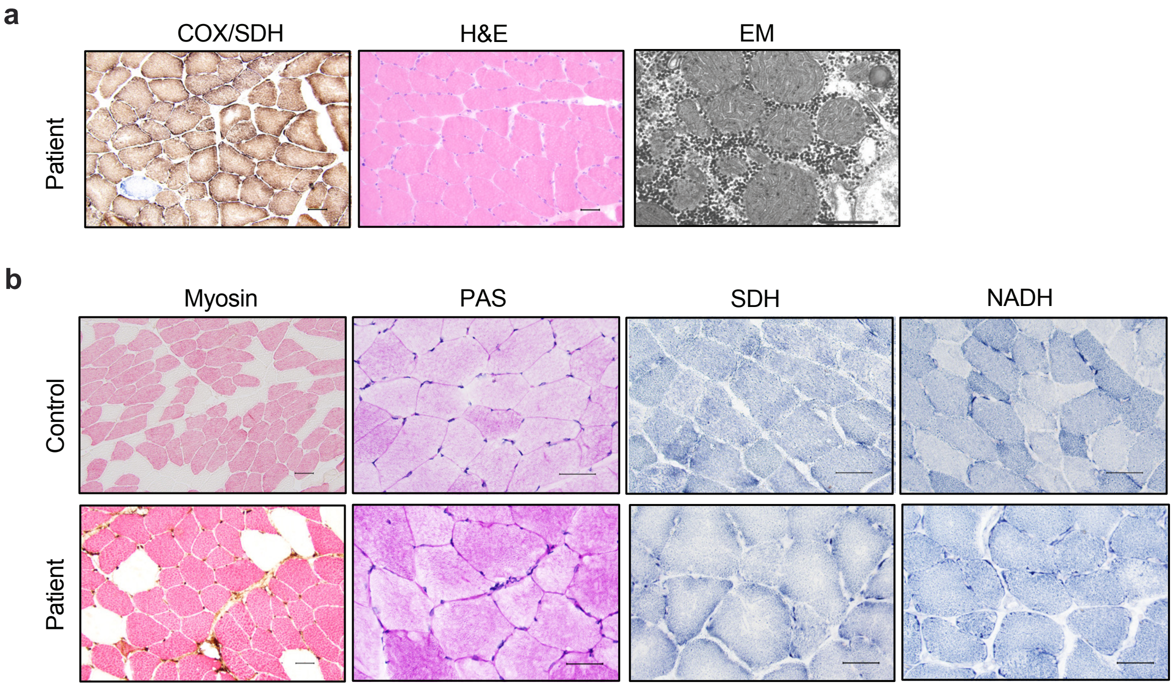

Supplementary Fig.5. Histochemistry and histology of skeletal muscle from the PARKIN-deficient patient. a. A skeletal muscle biopsy specimen from the *PRKN* patient stained with H&E for tissue morphology, COX/SDH for COX activity. High magnification transmission electron microscopy analysis was performed to define mitochondrial ultrastructure in skeletal muscle (Scale bar: 500 nm). **b.** Skeletal muscle biopsy specimens from the PRKN patient and an age- and gender-matched healthy donor were stained for myosin to discriminate between type 1 fibers (red) and type 2 fibers (white), Periodic acid schiff (PAS) staining to detect glycogen. Staining for succinate dehydrogenase (SDH) and nicotinamide adenine dinucleotide (NADH) to reveal myofibrillar architecture. (Scale bar: 50 μm).

Uncropped WB Fig. 1

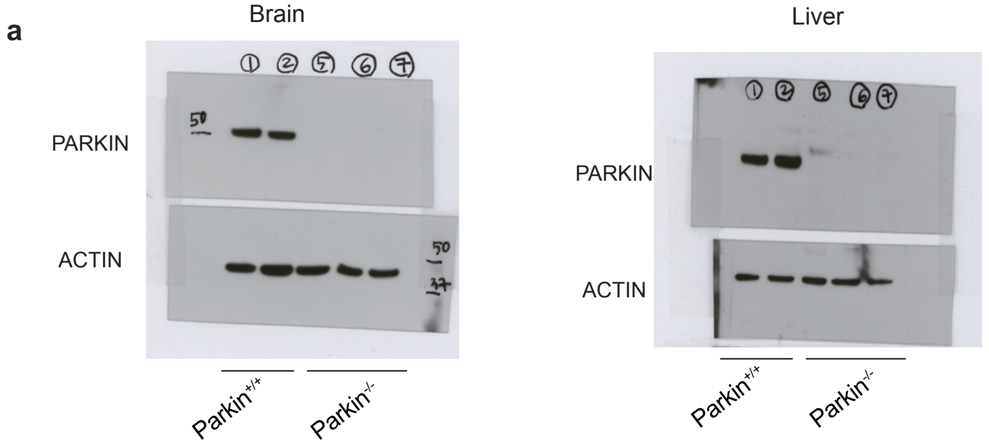

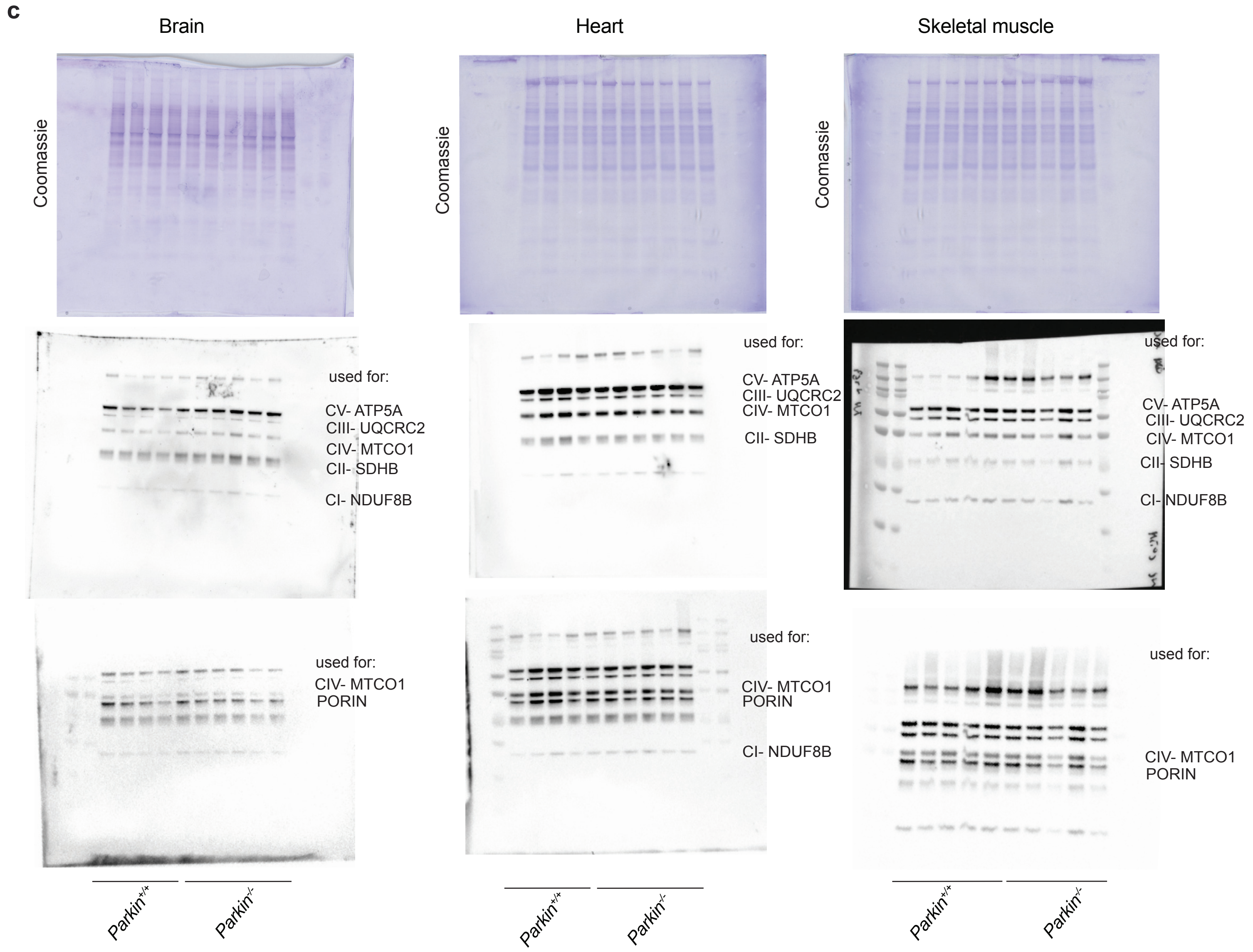

Uncropped WB Fig. 3

c

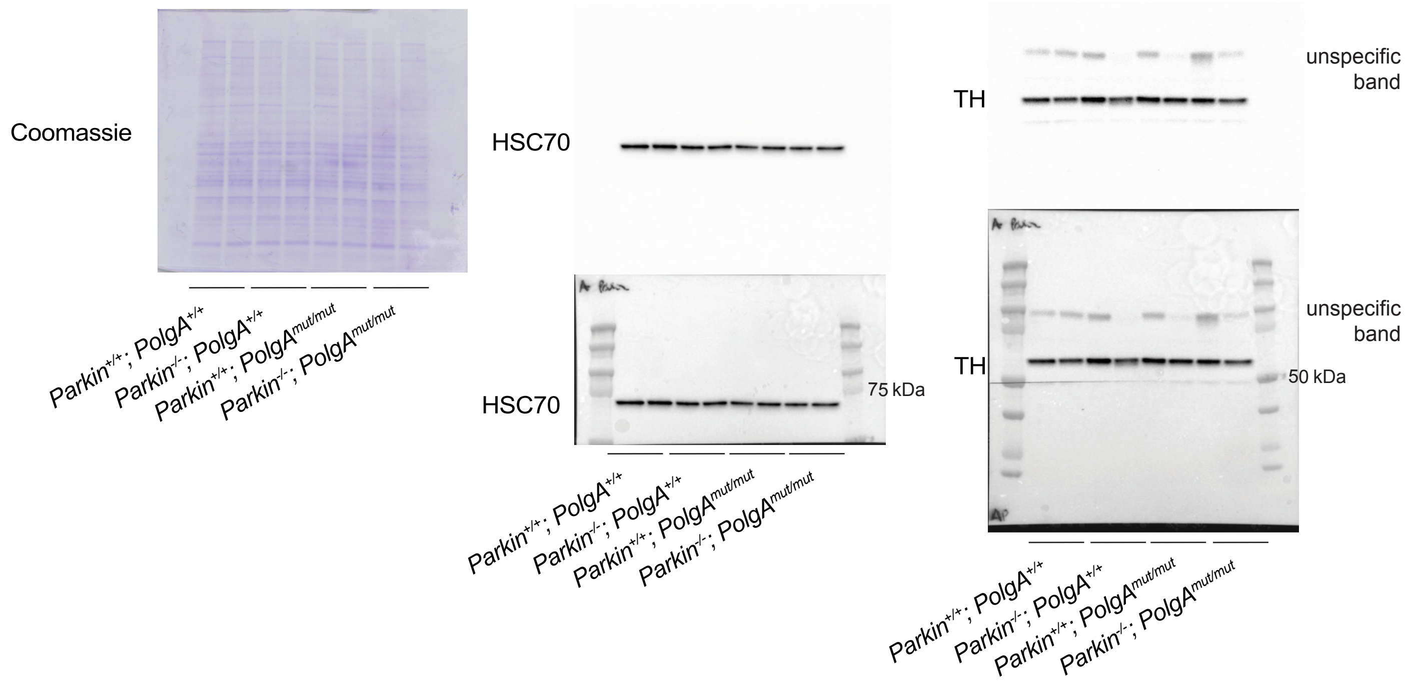

Uncropped WB Fig. 6

c

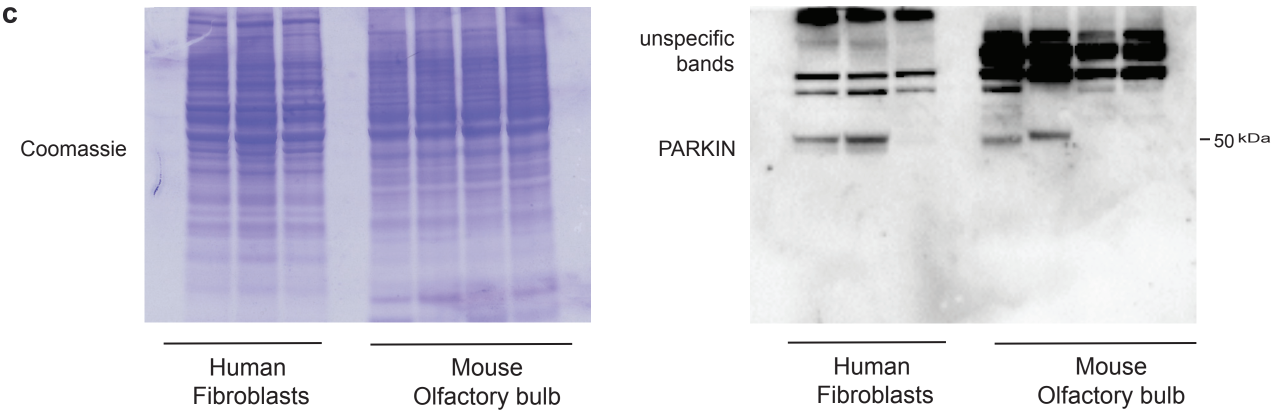

Supplement: Supplementary file 1 — Supplementary Figures [file 41531_2024_707_MOESM1_ESM.pdf]
