# Supplementary material for: PARKIN is not required to sustain OXPHOS function in adult mammalian tissues
Source: NPJ Parkinsons Dis. 2024 Apr 29;10:93. doi: 10.1038/s41531-024-00707-0 (PMC11058849; doi:10.1038/s41531-024-00707-0)
Supplement: Supplementary file 2 — Related Manuscript File [file 41531_2024_707_MOESM2_ESM.pdf]

nature portfolio

Corresponding author(s): Roberta Filograna
Nils-Göran Larsson

Last updated by author(s): Apr 3, 2024

Reporting Summary

Nature Portfolio wishes to improve the reproducibility of the work that we publish. This form provides structure for consistency and transparency in reporting. For further information on Nature Portfolio policies, see our Editorial Policies and the Editorial Policy Checklist.

Statistics

For all statistical analyses, confirm that the following items are present in the figure legend, table legend, main text, or Methods section.

n/a	Confirmed	
☐	☒	The exact sample size (*n*) for each experimental group/condition, given as a discrete number and unit of measurement
☐	☒	A statement on whether measurements were taken from distinct samples or whether the same sample was measured repeatedly
☐	☒	The statistical test(s) used AND whether they are one- or two-sided<br>*Only common tests should be described solely by name; describe more complex techniques in the Methods section.*
☒	☐	A description of all covariates tested
☒	☐	A description of any assumptions or corrections, such as tests of normality and adjustment for multiple comparisons
☐	☒	A full description of the statistical parameters including central tendency (e.g. means) or other basic estimates (e.g. regression coefficient) AND variation (e.g. standard deviation) or associated estimates of uncertainty (e.g. confidence intervals)
☐	☒	For null hypothesis testing, the test statistic (e.g. *F*, *t*, *r*) with confidence intervals, effect sizes, degrees of freedom and *P* value noted<br>*Give P values as exact values whenever suitable.*
☒	☐	For Bayesian analysis, information on the choice of priors and Markov chain Monte Carlo settings
☒	☐	For hierarchical and complex designs, identification of the appropriate level for tests and full reporting of outcomes
☒	☐	Estimates of effect sizes (e.g. Cohen's *d*, Pearson's *r*), indicating how they were calculated

Our web collection on statistics for biologists contains articles on many of the points above.

Software and code

Policy information about availability of computer code

Data collection	RNA Sequencing was performed on Illumina HiSeq 2500
Data analysis	Reads were aligned using Star v2.3.0 and filtered for uniquely mapping reads. Gene expression was calculated as read counts and as reads per kilobase gene model and million mappable reads (RPKMs) for each transcript in Ensembl release 75 using rpkmforgenes.<br>Hierarchical clustering was performed in R using the ward.D2 agglomerative method and the Pearson correlation-based distance measure.<br>Differential gene expression analysis was performed using DESeq2.

For manuscripts utilizing custom algorithms or software that are central to the research but not yet described in published literature, software must be made available to editors and reviewers. We strongly encourage code deposition in a community repository (e.g. GitHub). See the Nature Portfolio guidelines for submitting code & software for further information.

Data

Policy information about availability of data

All manuscripts must include a data availability statement. This statement should provide the following information, where applicable:

- Accession codes, unique identifiers, or web links for publicly available datasets
- A description of any restrictions on data availability
- For clinical datasets or third party data, please ensure that the statement adheres to our policy

The RNA-seq dataset generated and analyzed during the current study is available in NCBI's Gene Expression Omnibus with accession number GSE263057. Additional data generated or analyzed in this study are included in the published article and the supplementary information.

Research involving human participants, their data, or biological material

Policy information about studies with human participants or human data. See also policy information about sex, gender (identity/presentation), and sexual orientation and race, ethnicity and racism.

Reporting on sex and gender	We here have a single Parkinson's disease patient and a gender- and age- matched healthy donor. Both subjects are 52-year old females
Reporting on race, ethnicity, or other socially relevant groupings	does not apply
Population characteristics	does not apply
Recruitment	This patient was included in this study because she is carrying pathological variants in PRKN gene
Ethics oversight	The clinical part of the study was approved by the regional ethics committee in Stockholm and the Swedish ethical review authority (2019-04967). Informed consent was obtained from the patient and healthy donor.

Note that full information on the approval of the study protocol must also be provided in the manuscript.

Field-specific reporting

Please select the one below that is the best fit for your research. If you are not sure, read the appropriate sections before making your selection.

☒ Life sciences ☐ Behavioural & social sciences ☐ Ecological, evolutionary & environmental sciences

For a reference copy of the document with all sections, see nature.com/documents/nr-reporting-summary-flat.pdf

Life sciences study design

All studies must disclose on these points even when the disclosure is negative.

Sample size	Sample size and statistical tests are indicated in the methods section or figure legends. The sample size is based on previous experience. Usually, the sample size is n= 3-6 animals/experimental group. Due to variability, for behavioral analyses a larger cohort of animals was employed (n=7-17). Control and experimental mice have the same genetic background and usually litter mates, reducing variability.
Data exclusions	One single sample was excluded from the analysis of circulating plasma cytokines and chemokines (Figure 3G). This sample was outlier among the mtDNA mutator mice. In this mouse the levels of pro-inflammatory mediators were up to 20 times higher than in all the other animals with the same genotype, which may correlate with an ongoing acute immune response caused by an unidentified pathological condition. Thus, we have excluded this sample from our analysis. No data were excluded from other analyses.
Replication	As detailed in the figure legends, 2-5 independent biological replicas of all experiments were performed.
Randomization	Randomization of samples were not applicable in this paper. Neverthelss, experimental bias was addressed by preparing samples with different genotypes in parallel and using appropriate controls.
Blinding	Blinding was not used for most of the data collection or analysis, as the same researcher collected the samples, performed the experiments and analyzed the data. However, for the quantification of TH- positive neurons and mtDNA foci, blindness of the operator to the genotype of the analyzed cell/slice was used to ensure a more robust analysis.

Reporting for specific materials, systems and methods

We require information from authors about some types of materials, experimental systems and methods used in many studies. Here, indicate whether each material, system or method listed is relevant to your study. If you are not sure if a list item applies to your research, read the appropriate section before selecting a response.

Materials & experimental systems

n/a	Involved in the study
☐	☒ Antibodies
☐	☒ Eukaryotic cell lines
☒	☐ Palaeontology and archaeology
☐	☒ Animals and other organisms
☐	☒ Clinical data
☒	☐ Dual use research of concern
☒	☐ Plants

Methods

n/a	Involved in the study
☒	☐ ChIP-seq
☒	☐ Flow cytometry
☒	☐ MRI-based neuroimaging

Antibodies

Antibodies used	Primary antibodies:<br>PORIN (ab14734, Abcam)<br>PARKIN (ab15494, Abcam)<br>TH (MAB318, Chemicon)<br>HSC70 (sc-7298, Santa Cruz)<br>OXPHOS Rodent Antibody Cocktail (ab110413, Abcam) for NDUFB8 (CI), SDHB (CII), UQCRC2 (CIII), MTCO1 (CIV) ATP5A (CV).<br>anti-TH (213 102, Synaptic System)<br>anti-DNA (61014, Progen)
Validation	The antibodies have been validated by the manufacturer, validation details and relevant publications are detailed in their respective websites.<br>PORIN (ab14734, Abcam)<br>hhttps://www.abcam.com/products/primary-antibodies/vdac1porin--vdac3-antibody-20b12af2-ab14734.html<br><br>PARKIN (ab15494, Abcam)<br>https://www.abcam.com/products/primary-antibodies/parkin-antibody-ab15494.html<br><br>TH (MAB318, Chemicon)<br>https://www.merckmillipore.com/SE/en/product/Anti-Tyrosine-Hydroxylase-Antibody-clone-LNC1,MM_NF-MAB318?ReferrerURL=https%3A%2F%2Fwww.google.com%2F<br><br>HSC70 (sc-7298, Santa Cruz)<br>https://www.scbt.com/p/hsc-70-antibody-b-6<br><br>OXPHOS Rodent Antibody Cocktail (ab110413, Abcam)<br>https://www.abcam.com/products/panels/total-oxphos-rodent-wb-antibody-cocktail-ab110413.html#:~:text=Total%20OXPHOS%20Rodent%20WB%20Antibody%20Cocktail%20ab110413%20is%20an%20optimized,mouse%20mitochondria%20by%20western%20blot.<br><br>anti-TH (213 102, Synaptic System)<br>https://sysy.com/product/213102<br><br>anti-DNA (61014, Progen)<br>https://www.progen.com//anti-DNA-mouse-monoclonal-AC-30-10-lyophilized-purified/61014

Eukaryotic cell lines

Policy information about cell lines and Sex and Gender in Research

Cell line source(s)	Human fibroblast lines were obtained from the Center of inherited metabolic diseases at Karolinska University Hospital. Two control cell lines with normal mitochondrial biochemistry was selected for this study. Patient fibroblast line was obtained from a subject affected by Parkinson's disease caused by PRKN gene mutations.
Authentication	Human fibroblast lines are not authenticated. Fibroblast biopsies and establishment of fibroblast cultures is part of clinical routine at the Center of inherited metabolic diseases at the Karolinska University hospital for more than 30 years. The procedure is described in the following publication: https://ng.neurology.org/content/7/2/e566.
Mycoplasma contamination	All cell lines tested negative for mycoplasma infection.
Commonly misidentified lines<br>(See ICLAC register)	No commonly misidentified cell lines were used in this study.

Animals and other research organisms

Policy information about studies involving animals; ARRIVE guidelines recommended for reporting animal research, and Sex and Gender in Research

Laboratory animals	All mice were on the C57BL/6N genetic background. Experiments were performed using three different mouse models and respective controls at different time points (15 months for Parkin whole body KO; 36 weeks for Parkin KO; mtDNA mutator; 5, 20, 40 60 weeks after injection for tamoxifen-inducible Parkin KO). In each experiment, both male or female mice were employed.
Wild animals	No wild animals were used.
Reporting on sex	No findings were applied to one gender.
Field-collected samples	No samples collected from the field.
Ethics oversight	All animal procedures were conducted in accordance with European, national, and institutional guidelines and protocols were approved by the Stockholms djurförsöksetiska nämnd, Sweden and by the Landesamt für Natur, Umwelt und Verbraucherschutz Nordrhein–Westfalen, Germany. Animal work was performed in accordance with the recommendation and the guidelines of the Federation of European Laboratory Animal Science Associations (FELASA).

Note that full information on the approval of the study protocol must also be provided in the manuscript.

Clinical data

Policy information about clinical studies

All manuscripts should comply with the ICMJE guidelines for publication of clinical research and a completed CONSORT checklist must be included with all submissions.

Clinical trial registration	This study did not involve clinical trial
Study protocol	No study protocol needed
Data collection	The PD-patient involved in this study was recruited because she carries a known mutatation in PRKN gene.<br>Assessment of presynaptic dopamine transporter was done using DaTSCAN SPECT. Genetic and biochemical analyes were conducted on skeletal muscle biopsy from the anterior tibial muscle.<br>Additional analysis were performed on patient fibroblast.<br>The clinical part of the study was approved by the regional ethics committee in Stockholm and the Swedish ethical review authority (2019-04967). Informed consent was obtained from the patient.
Outcomes	DaTSCAN SPECT examination showed major loss of the dopamine transporter (DAT) in the basal ganglia<br>Genetic analysis identified the presence of a known and a novel PRKN pathogenic variant which together abolish the expression of PARKIN.<br>In skeletal muscle, there were no signs of mitochondrial myopathy and OXPHOS dysfunction.

Plants

Seed stocks	This study did not involve the use of plants
Novel plant genotypes	This study did not involve the use of plants
Authentication	This study did not involve the use of plants
